# On the Physiology and Pathology of the Brain
*We publish this paper without expressing our concurrence in all the views entertained by the author.—Ed.


**Published:** 1852-01-01

**Authors:** Joseph Lalor

**Affiliations:** PHYSICIAN TO THE KILKENNY LOCAL LUNATIC ASYLUM, ETC.


### ON THE PHYSIOLOGY AND PATHOLOGY OF THE BRAIN.*

BY JOSEPH LALOR, M.D., PHYSICIAN TO THE KILKENNY LOCAL LUNATIC ASYLUM, ETC.

There can be no doubt that structural lesions of the brain, and abnormal conditions as to the quantity of the blood circulating therein, and the rate and mode of its circulation, will disturb the cerebral functions in various ways. But, in many cases of nervous and mental disturbances, and more especially of mania, and allied disorders, no such structural lesions or abnormal conditions of vascularity exist. Moreover, the derangements of intellect which coincide with structural lesions of the solid portions of the brain (when those lesions are uncomplicated) and with vascular congestions of that organ, appear, at least, in general, to amount to no more than a weakness, a defect of the faculties of the mind; such as a slowness of comprehension, lethargy, insensibility, or that species of incoherence, which is merely a want of power to continue the attention to one point, or to one train of ideas. In Andral's *Clinique*, there are described the *post mortem* appearances, in twenty-four fatal cases of affections of the membranes of the brain, consisting of tumours of the dura mater, sanguineous effusions between the dura mater and arachnoid, and of inflammations of the arachnoid, the dura mater and the pia mater, in various stages and degrees of intensity. Delirium occurred in seventeen of those cases during life, and although the precise character of the delirium is not expressly stated, yet from the notice of the presence of other symptoms, showing a want of vital energy, and of the alternation, coincidence, or succession, of coma and stupor, there can be little doubt that the character of the accompanying mental affection was of that asthenic description already described. But inflammations of the membranes admittedly produce more frequent, better marked, more sthenic, and more violent mental disturbances, than inflammations of the substance of the brain. Indeed, Lallemand undertakes to prove, that delirium is never observed in inflammations of the substance of the brain, which are exempt from complication, and that this symptom belongs especially to inflammations of the arachnoid. In seventy-five fatal cases of affections of the

* We publish this paper without expressing our concurrence in all the views entertained by the author.—Ed.

brain in adults, which are related in Abercrombie's work on diseases of the brain, the *post mortem* appearances are described, consisting of structural lesions of various forms and degrees of intensity. Delirium was absent during life in sixty of those cases, and present only in fifteen. In inflammations of any other important organ, as well as of the brain, delirium will frequently arise. A paper from the pen of Sir Everard Home, in the twenty-fourth volume of the "Edinburgh Review," exhibits, in a very striking and circumstantial manner, the extent of injury which the brain may sustain, without any suspension of its faculties; and the perusal of this paper can scarcely fail to leave a strong impression on the mind, that considerable structural lesion of the brain may exist with little or no mental disturbance. On the other hand, a careful and impartial investigation of the results of *post mortem* examinations will, I am convinced, tend to show, that, where the intellect has deviated most permanently and obviously, from the healthy standard; as in mania and its allied disorders, there is frequently found to be no structural lesion whatever of the solid portions of the brain. When such lesions have been found to exist, they have been most frequently of such slight extent, and of such recent origin, as to shew that they have been, not the cause of the violent mental disturbance, but rather accidental coincidences or consecutive alterations. In a review of recent publications on insanity, which appeared in the "Dublin Medical Journal for November, 1850," the reviewer observes, that the pathology of insanity is very obscure, and throws little light on the disease, and he quotes from a recent work of Sir Alexander Morison's this observation:—"That Esquirol opened the bodies of no fewer than three thousand persons who died in an insane state, and yet he says, that he would be at a loss to tell what is the precise part of the brain diseased. Greding, Pinel, and Haslam have also drawn nearly similar conclusions." When such unsatisfactory results have been obtained from such an extensive series of investigations, confined to the solid portion of the brain, which is admitted, at the present day, to be the material organ of the mind, I trust that I shall be excused for endeavouring to invite the attention of the profession to a closer consideration of the influence which the quality of the blood appears to exercise over the functions of the brain, as offering at least a prospect of throwing light on a question eminently obscure and difficult.

In the following observations, it is intended to adduce some facts, authorities, and arguments, which have occurred to me, indicating the existence of a constant relation between the chemical and physical constitution of the blood circulating in the brain, and the powers, character, perfection, and imperfection of the cerebral functions.

Richerand, in his physiology, attributes some of the moral qualities of men and animals, such as courage, to the activity with which the heart propels the blood towards all the organs, and he says, "that every being that is feeble is timorous, and shuns danger, because an inward feeling warns him that he does not possess sufficient strength to resist it." He adds, "It may be observed that some animals, as the turkey-cock, and the ostrich, possess less courage than the least birds of prey; that the ox has less than the lion, and other carnivorous animals. What has been said applies to the relative and not to the absolute size of the heart. Now, though the heart of a hawk be absolutely smaller than that of a turkey-cock, it is nevertheless larger in proportion to other parts of the animal. Besides, birds of prey, like other carnivorous animals, in part owe their courage to the strength of their weapons of offence." Richerand's observation as to the difference of courage between herbivorous and carnivorous animals is well known to be true as a general rule; and he might have added many other differences in their moral qualities, as the greater proneness of carnivorous animals to violent passions, their indocility and untameableness, and, on the other hand, the greater mildness and docility of herbivorous animals. For my part, I believe that a rigid examination of the question would show a more constant relation to exist between the quality of the blood in herbivorous and carnivorous animals, and the acknowledged differences in their dispositions, than between the relative size of their hearts, or the strength of their weapons of offence, and those dispositions, as suggested by Richerand. At all events, great differences have been ascertained to exist in the composition of the blood of those animals, consisting, amongst others, in a greater proportion of globules and a less proportion of water in the carnivorous than in herbivorous animals. The following quotations from M. Lecanu's essay on the blood not only establish the fact of this difference in the quality of the blood in those classes, and also in others where analogous differences in disposition exist, but also go far to show the efficiency of such difference in the composition of the blood, as a cause to produce such difference in disposition:—"The proportion of globules would seem to serve as a measure of the vital energy. This general result is of extreme importance, when we remember what MM. Prevost and Dumas have taught us of the totally different action exercised upon the nervous system by the serum which scarcely excites it, and by the globules which excite it violently. By a singular coincidence, every cause which tends to diminish the mass of the blood seems to tend, at the same time, to diminish the relative proportion of the globules, whilst it increases that of the water in such a manner, that the influence of those causes has for its results to produce both the lesser fulness of the blood-vessels, and the impoverishment and fluidity of the blood which they contain. In women the uterine losses, and in the two sexes blood-letting, and a diet of solid aliments produce this double effect in a remarkable manner. In a previous paragraph Lecanu had stated, on the authority of Prevost and Dumas, that the proportion of globules was greater in carnivorous than in herbivorous animals." It is further stated by Richerand,* as a well-known fact, that "the greater or less degree of the vicinity of the heart and brain gives a tolerably just measure of the intellect of man and the instinct of the lower animals, and that the disproportionate length of the neck has ever been a mark of stupidity." The simple explanation of this fact (assuming it to be true), would appear to be the more impure or carbonised condition of a fluid so delicate and so susceptible of change as the blood, owing to the greater length of its passage from the heart to the brain.

There are reasons for supposing that the relative perfection of the intellect, in man and animals, is in some measure dependent on the quality of their blood. I am fully aware that the majority of physiologists give a different explanation of this matter, and that they generally agree in the view put forward by Müller,† viz., "Corresponding with the development of the intellectual faculties in the different classes, we meet with very great differences in the form of the brain, which undergoes a gradual increase of size, from fishes up to man, with the development of their intellectual faculties, which in the animal scale is dependent on the size of the brain. But those propositions of Müller's are not strictly in accordance with fact, and we do not find that the increase in size in the brain of animals is exactly accordant with the development of their intellectual faculties. The scale of this increase in size is by no means regularly graduated, but runs up and down in an irregular manner, so that we find a whole species of an inferior class of animals, whose brains are more voluminous than those of another species in a superior class, and that we also find a greater development of the intellectual faculties in individuals of species with smaller brains than in others of species with larger brains, whether the size of the brain be taken absolutely or relatively to the size of the rest of the body. Bostock‡ remarks, that "all those comparative observations (such as we have quoted above from Muller) are deserving of attention; but we might, *à priori*, expect the powers of the nervous system to depend, at least, as much upon the

* Richerand. By Copeland. P. 330.

† Muller. By Baly. Pp. 808, 815, and 816, vol. i.

‡ Physiology. 4th edition. P. 168.

perfection of its organization as upon its mere bulk." However, the perfection of the organization of the brain, and its adaptation to the purposes intended, must depend on the perfection of its fluid as well as of its solid constituents. The quantity of the blood circulating in the brain, the mode and rate of its circulation, and its composition, are most probably all causes affecting the development and perfection of the cerebral faculties, as well as the mere volume of the organ—whether considered as a whole or relatively to its parts—and molecular structure. A consistent and perfect explanation of many phenomena in health, and disease, can be afforded only when due notice of the quality of the blood is included, with other considerations, as forming a portion of the physiology and pathology of vital action, whether mental or bodily. I repeat, that great differences exist in the constitution of the blood in different species of animals, and in different individuals of the same species; some of which have been already stated, and others will now be considered. Leuwenhœck has pointed out great differences in the form and size of the globules of the blood in animals of different species; and even previous to his time the division of animals into white-blooded, and red-blooded, cold-blooded, and warm-blooded, showed that great differences had been considered to exist between the qualities of the blood in different species of animals. Latterly a number of scientific men have contributed much to increase our knowledge of this subject, and have made various observations and analyses of the blood of several animals, and of man, in health and disease. Amongst those, M. Lecanu, from the results of experiments made by himself and by M. Denis, asserts, that the effect of bad and innutritious diet is to diminish the quantity of globules, and to increase the quantity of water in the blood.

MM. Prevost and Dumas have shown by analysis, that the proportion of globules is greater, and the proportion of water less in birds, than in other animals, and as already remarked in carnivorous, than in herbivorous animals, and on the contrary that the proportion of water is greater, in animals with cold blood, than in animals with warm blood. Lecanu has also proved that the proportion of iron differs in the blood of different species, and of different classes of animals. So far then as qualitative analyses have gone, they have proved the existence of differences, in the quality of the blood, corresponding with differences of classes, and of species of animals, of their general mode of nourishment, of particular alterations of diet, of the nature and perfection of their respiratory functions, &c. Those differences in the qualities of the blood in animals are not less marked, than the differences in the volume of their brains, its structure, or its vascular peculiarities; whilst the difference, for instance, between the nerve-exciting power of the globules and the serum, indicates that differences in the quality of the blood may produce differences in disposition, temper, intellect, &c. Naturalists, and the keepers of menageries, have long since remarked that the ferocity and thirst for blood, of beasts of prey, is violently excited by a meal of raw meat or of blood. The courage and spirit of game-cocks and of race-horses are also increased by the use of particular articles of solid or of liquid aliment; and this moral change is often produced in a space of time too short to allow of the supposition that it is owing to any alteration in the solid structure of any organ. Again, very perceptible differences arise in the physical properties of the blood, sometimes according to the medium through which respiration is carried on, and sometimes according to the air or gases which are respired. The various processes of nutrition, and of secretion, materially affect the composition of the blood, and as those processes are more or less numerous and more or less perfect, so must the quality of the blood differ both in individuals and in species. In fact, I am led to think, from some reflection on the subject, that a careful analytic investigation would show at least as regular a gradation in the blood as in the solids, throughout the animal kingdom, and that in individuals also their blood would be found, by such an inquiry, to differ either coincidently with or consecutively to original or acquired differences of organization, or as they were placed in circumstances favourable or unfavourable to the performance of their functions. Certainly there is not a single function of the animal body which does not appear to affect the composition of the blood, and to be affected by it. Of all the animal functions, that of respiration exerts the most direct and largest influence on the blood. In proportion to the perfection of the function of respiration we constantly observe a proportionate energy, and perfection of the intellectual faculties, other circumstances being equal; and so, in like manner, as to the effects on the intellect of imperfect respiration. The results are analogous, whether the perfection or imperfection of the respiratory process arise from the perfection or imperfection of the organ by which it is effected, the nature of the gas respired, or the nature or conditions of the respiratory medium as to circumstances favourable or otherwise to the respiratory process, such as light, moisture, temperature, &c. Thus analogous impairments of the vital energy and of the intellectual faculties, arise when carbonic acid gas loads the blood; whether the excess of the gas arises from its direct inhalation, from the want of its removal by the natural processes, from the deficiency of oxygen gas in the air respired, from the imperfection of the circulating organs, as in *morbus cæruleus*, or from any other cause, provided the other circumstances which might modify those results, remain equal. Thus also we find the energy and irritability of birds to bear a proportion to the amount of their respiration, which is determined chiefly by the peculiarities of their respiratory organs. "Every one knows," says Cuvier,* "the varied industry which birds exhibit in the construction of their nests, and the tender care which they take of their eggs and young; it is the principal part of their instinct. With regard to the rest, their rapid passage through different regions of the air, and the intense and continued action of that element upon them, renders them pre-sensible of the variations of the atmosphere to an extent of which we can have no idea, and from the most ancient times has caused to be attributed to them, by superstitious persons, a power of announcing future events. They are not devoid of memory, and even imagination, for they dream, and every one knows with what facility they may be trained, taught various services, and to retain airs and words." Yet the volume of the brain in birds, as a class, is inferior to that of mammals, and chiefly depends on the tubercles analogous to the corporoa striata. With such brains, and with less energetic and less perfect respiratory and circulating apparatus, would the intellect or instinct of birds rate so high?

"The three classes of oviparous vertebrates," says Cuvier,† "differ very much from each other in their quantum of respiration, and in all that relates to it; viz., the power of movement, and the energy of the senses. Again, ‡ "As respiration imparts the warmth to the blood, and the susceptibility of the nerve-fibre, reptiles have cold blood, and their aggregate muscular energy is less than in the mammalia, and much less than in birds. Hence their movements can scarcely be performed otherwise than by crawling, and swimming; their habits are generally sluggish, their digestion excessively slow, their sensations obtuse; and, in cold or temperate climates, they pass nearly the whole winter in a state of lethargy. The amount of respiration in this class is not fixed, as in the mammalia and birds; but it varies, according to the relative proportion of the diameter of the pulmonary artery, as compared with that of the aorta. Thus tortoises, and lizards, respire much more than frogs; hence the differences of energy, and sensibility, are very much greater than those between one mammal and another. It surely will not be denied that the intellectual powers of animals may be influenced by the amount of their respiration, when it is admitted that this influences their vital energy, the susceptibility of their nerve-fibre, to the energy of their senses, digestion,

* Animal Kingdom. By Blythe, &c. pp. 154 and 161.

† Idem, p. 153. ‡ Idem, pp. 267, 268.

and sensations; and when it is also admitted that the imperfection of the respiration renders the movements of certain classes of animals sluggish, and reduces them, on a slight decrease of temperature, to a state of lethargy. But the results, on the functions of the brain, arising from differences in the perfection or imperfection of the respiratory process, are (as already stated) analogous; although the causes giving rise to those differences may be very different, or even opposite, as to their effects on the animal economy, other than as regards the respiratory process. It follows that it is the respiratory process (the action of which is on the blood) which influences the functions of brain, and not the effect of sympathy with some other organ (on which perhaps the defect in respiration depends, as in *morbus cæruleus*); nor on accessory circumstances (as those might be supposed to act otherwise than by their influence on the respiration, and thence on the blood). Another presumptive proof of the influence of the quality of the blood on the intellectual faculties of animals, may be drawn from the change of character produced in them by change in geographical position; as the chief accidents of geographical position are such as most probably act through the large influence which they exercise on the composition of the blood. Those are temperature, state of the atmosphere as to its hygrometric, or electric condition, diet, &c. &c. It is well known that animals, like plants, affect a certain geographical zone, out of which the species indigenous to that zone do not come to perfection, and in many instances will not live at all. When far removed from their natural geographical habitat, animals lose their spirits, the activity of their intellect and of their character becomes altered; they often will not continue their species; they become liable to disease, pine, and die. Changes, then, from their natural geographical habitat, effect changes in the economy of animals, as to the energy of their intellectual faculties, their habits, their dispositions, their passions, and their sexual propensities. The quality of their blood, too, must be affected by the alterations in external circumstances produced by change in geographical position, and deeply affecting the functions, especially of the respiratory organs, adapted to a different zone. Is there any other physical change, which does occur, or is likely to occur, in animals, from change of geographical position? The same train of argument might be pursued, as to the effects of domestication on animals; but there is so much difficulty in separating what is the result of physical causes from what is the result of education, that I will not enter into the subject more than to remark that the difference in sexual propensities, between the wild and the tame pigeon, for instance, do not appear to have any causes to explain them, other than those differences in the external physical circumstances of the two varieties, which act on the blood, and through it on the nervous centres.

An extraordinary instance of the effect of food on the entire organization of the larva of an animal, to such a degree as to alter the sex, and thus to modify the whole structure of the intellect in the future animal, is presented in the bee species, of which the workers may be transformed into mother bees, if, while larvæ, and during those first days of their existence, they receive a peculiar nourishment, such as is alone given to the larvæ of future queens. It is only reasonable to suppose that the food acts in this instance as it must be supposed to do in ordinary circumstances—viz., through its influence on the composition of the blood. In the human species, the proportion of male to female births is greatly influenced by the condition of the parents as to circumstances which either have been ascertained experimentally to affect the constitution of the blood, or which may be inferred, rationally, and from analogy, to do so. Thus it would appear, from the results obtained by M. Quetelet himself, or by other statistical authorities quoted by him,* that "the relative ages of the parents, their employments, their condition as to physical constitution, supply of food, &c., their residence in town or country, exert a regulating influence on

* Quetelet on Man. Translated by Chambers.

the proportion of the sexes in a given number of births. It has been already shown that the quality of the blood is affected by some of the circumstances mentioned above—as food; and, hereafter, it will be shown to be affected by others—as age. But, in the mean time, it may be observed, that there is not one of those circumstances which we can well suppose to be without influence on the constitution of the blood; and I believe it would be impossible to point out any other common physical effect on the animal economy, in which they can all be supposed to agree. It can scarcely be necessary to remark that, what plays a part in determining the future sex, must likewise play a part in determining the future moral and intellectual qualities of an individual organism. The transmission of hereditary mental qualities, and of peculiarities of physical organization, from the male parent to his offspring, presents an instance of the action of a fluid or solid matter; if not identical in its nature, yet of a character at least as peculiar, and as difficult of comprehension, as would be the production of ideas by the action of the blood on the brain, under the superintendence of an immaterial mental principle, should such a theory be adopted. "The semen," says Müller,* "is not merely a stimulus for the fructification of the egg, for it impregnates the eggs of batrachia and fishes, out of the body; and the form, endowments, and even tendencies to disease, of the father, are transferred to the new individual. The semen, therefore, although a fluid, is evidently endowed with life, and is capable of imparting life to other matter." Again,† "The semen and germ must contain, not only the vital principle, but also the mind of the new being in a latent state." Again,‡ "Observations have shown, that the fecundation following the union of the sexes results from the direct action of the semen on the ovum. It is equally certain that fecundation does not depend on any influence of the entire male organization, but on the semen alone." Those doctrines of Müller are fully borne out by the facts and arguments which he adduces to support them, and establish for the semen that influence which he attributes to it. But there is a phenomenon more curious than the transmission of qualities from the male parent to his direct offspring through the semen. It is that the peculiarities of a male animal, that has once had fruitful intercourse with a female, are more or less distinctly recognised in the offspring of subsequent connexions of that female with other males. Such instances commonly occur among the lower animals, and several are cited in the appendix to Combe's "Constitution of Man." Similar facts are recorded, also, in a paper from the pen of Dr. Harvey, of Aberdeen.§ Dr. Harvey sets forth a theory to account for those long-observed and well-established facts, which has been put forward by Mr. M'Gillivray, a veterinary surgeon, and which theory Dr. Harvey supports. This theory is as follows:—"When a pure animal of any breed has been pregnant to an animal of a different breed, such pregnant animal is a cross for ever after; the purity of her blood being lost, in consequence of her connexion with a foreign animal." Dr. Harvey explains the loss of the purity of the blood in the following manner:—"In the same manner as the small-pox virus may pass unaltered from the mother to the child in her womb, and produce in it actual disease, so also, constitutional peculiarities, derived to the fœtus from the father, and inherent in its blood, may be imbibed with the blood by the mother. When we reflect on the length of time, during which the connexion between the mother and foetus is kept up, and the amount and activity of interstitial change going on in the system of the fœtus; the large quantity of fœtal blood that must eventually be taken into the vessels of the mother; and the probability, that the peculiar matter imparted by the male parent to the ovum, at the moment of impregnation (be its nature what it may, and its quantity never so infinitesimal), assimilates most of the fœtal blood to itself; it does not seem too hard to be believed, that the blood and constitution generally of the mother

* Müller. By Baly. P. 144, vol. i. † P. 820. ‡ P. 1489, vol. ii.
§ See Med. Press (Dublin), Oct. 10, 1849. Copied from Ed. Month. Journal.

may thereby become so imbued with the peculiarities of that parent as to impart them to any offspring she may subsequently have by other males." Dr. Harvey cites instances of analogous phenomena in the human species, showing the transmission of qualities from the male who has had the first fruitful intercourse with a female,—not only to the offspring of that intercourse, but to the offspring resulting from subsequent connexions with a different male, and even to the mother herself. Those instances in the human species he explains by a similar theory. The whole scope of this ingenious and interesting paper is strongly in support of the views which I advocate, and tends to prove that the influence of the semen, not only on the physical, but also on the mental organization of the fœtus, and of the mother, is realized through the medium of the blood. In some results arising from the transfusion of blood, and in the limitations and laws fixed by nature to regulate the fruitfulness of intercourse between animals of different genera, &c., we have a series of parallel facts which appear to have an important bearing on our present subject, and to be capable of rational explanation only on a common principle, having reference to the constitution of the blood. It is known that the blood-globules have different dimensions in the different animal species, and similar forms and dimensions in the same species; and also that revivification is produced in an animal bled to syncope, by the transfusion into his veins of the blood of an animal of the same species. But a deadly effect is produced by the transfusion of the blood of some classes of animals, into the system of animals of other classes; as of the blood of mammalia into the veins of birds. "The deadly effect in those cases," says Dr. Bischoff,* "is in some way connected with the fibrine. The principle which renders the blood of one class of animals injurious to another class, is not the vivifying principle of the blood, which might be supposed to be peculiar to each individual class, and deadly to others; for the blood, when deprived of its fibrine by stirring, has still the effect of perfectly restoring the animal from which it was taken, although the latter be reduced by loss of blood to syncope, or apparent death; but it is an important fact, that when blood, thus deprived of its fibrine, is injected into the veins of an animal of a different class, reduced to a similar state of syncope, no revival takes place; the animal dies. Thus, we have several series of phenomena coincident with certain ascertained qualities of the blood: firstly—the limitation of the fruitfulness of connexions between animals to those between animals, the globules of whose blood are similar, or nearly so; secondly—the revivifying powers of blood, deprived of its fibrine, on the same animal from which it was taken; thirdly—the non-revivifying power of blood, deprived of its fibrine, on an animal of a different class; fourthly—the otherwise innocuous influence of blood so treated, even on animals of a different class; fifthly—the poisonous effects of the blood of some classes of animals of which the fibrine has been retained, when introduced into the system of animals of other classes; sixthly—many of those poisonous effects are manifested on the nervous centres, and on the intellectual faculties. And, in an abstract view, we see the power of the continuation of certain types and forms of physical and mental organization amongst animals, the power of the continuance of individual animal life, and healthy nervous and intellectual action, coincident with certain qualities of blood.

We find it stated in "Bostock's Physiology," that Haller made a calculation, from which he concluded that one-fifth of all the blood sent out from the left ventricle is carried to the head, although the weight of the brain in the human subject is not more than one-fortieth of that of the whole body. Whether we adopt this calculation, or reduce the quantity of blood to one-tenth, according to Monro, or take even a lower estimate, we cannot but be struck with the very large supply of blood which the brain receives in proportion to the rest of the body; and hence we must be led to inquire for what purpose is such a large

* Müller, p. 141, vol. i.

supply provided, and the freedom of its circulation secured by the peculiar mechanism of the cerebral blood-vessels? The processes of secretion and renovation performed in the brain are trivial, and manifestly insufficient to require a supply so large, and a circulating apparatus so peculiar. Indeed, wounds and injuries of the brain are repaired slowly, in comparison with those of other parts of the body; and it may be doubted if true nervous matter is ever reproduced. The molecular augmentation of the brain, evidenced by its increase in size and density from youth to manhood, is so slow, trivial, and limited in its duration to a certain period of life, as not to require provisions so ample and so permanent. There remains only the supposition, that this large supply of blood, and the peculiarities of the circulating apparatus, subserve the purposes of the peculiar functions of the brain. Can we suppose that it is a matter of indifference what is the quality of that fluid whose circulation in the brain with such freedom, in such quantity, and with such constancy, is thus carefully and amply provided for? The brain also receives the blood in a highly arterialized state. "There are some organs," says Bostock,* "more particularly the brain, the spinal cord, and the organs of sense, which are, at least, much less plentifully supplied with absorbents than the other soft parts; indeed, it may be doubted if we have any unexceptionable evidence of their having been seen in those organs." This total or partial absence of lymphatics in the brain, and the scantiness of its nutrient and secreting functions, are peculiarities which appear to have a close and common bearing on the subject under review, and which should be considered not only in the aspect as they affect the composition of the blood, but also as they afford grounds for reasoning on the mode of action of the blood upon the brain, if the existence of some action on the part of that fluid be presupposed. Supposing the office of the lymphatics to be the conversion of the solids of the body back again to the fluid form, and their return into the general circulation (to be eliminated therefrom by the different excreting organs, or purified by the respiratory organs), it appears to follow, as a consequence of the absence of lymphatics from the brain, that this process of elimination or reparation is wanted therein, and hence also, that the brain is a permanently organized body. The nervous matter of the brain occupies the same position between the termination of the arteries and the commencement of the veins as is occupied by the secreting and nutrient vessels of some other organs; and the mass of the blood circulating through the brain is converted from arterial into venous, whilst it permeates the medullary molecules. But as only a small portion of this large supply of blood is wanted for any process of secretion, molecular renovation, or growth, it follows that the arterialization of the remaining larger portion must be otherwise accounted for. There remain only the peculiar functions of the brain to account for this conversion, and as the blood in the jugular veins is similar, or nearly so, in quality with that in the vena cava, for instance, it follows that an analogous reaction occurs in the parts from which the blood is returned to those different veins. Hence it would appear that the functions of sensation, perception, motion, &c. are developed just at the point where the arterial is being altered into venous blood, or at the precise point where secretion and nutrition take place in other parts of the body. It seems natural to conclude that this alteration in the blood is not only contemporaneous with, but consequential on, the function, in the one case as in the other; and that the same chemical action which, in the one case, is followed by a new material product, is, in the other, connected, in some way, with the development of sensation, &c. From the commencement of fœtal life to the termination of independent existence in man, variations in the condition of his cerebral powers and qualities are found constantly to coincide with variations in the constitution of the fluid blood circulating in the system. "The excitement," says Müller,† "of certain organic states of the brain, by the bright

* Note to p. 603 of Physiology, 4th edition. † P. 1387.

scarlet, aerated blood, is a necessary condition for the action of the mind. Hence, the abstraction of blood in large quantities produces syncope, and loss of consciousness."

"The blood of the fœtus, arterial and venous, is stated," by Müller,* "to differ in no respect from the venous blood of the adult." It is impossible to reconcile such a conclusion with the opinion generally received, and in which Müller himself joins, that the placenta performs an office supplementary to the lungs. As the two opinions are irreconcilable, let us see which is the correct one. The anatomy of the placenta, the venous condition of the embryotic blood, and the low power of generating heat in the new-born animal, would lead to the conclusion that the placenta performs an office the very reverse of that assigned to it; and Muller himself says, "that in the fœtus of mammalia the necessity for the aeration of the blood seems wholly wanting."† Certainly, whilst the blood enters the maternal placenta in an arterial condition from the uterine arteries, none passes out of the placenta except in a venous condition, whether on the one hand that which goes through the umbilical vein, after imbibition from the maternal placenta, into the fœtus; or, on the other hand, that which returns into the maternal system by the uterine veins. In fact, from what occurs at the moment when the blood of the fœtus at birth has been exposed to the influence of the atmospheric air—viz., the simultaneous development of motion, sensation, perception, hunger, and other appetites, we must suppose that the same results would follow from the circulation of arterialized blood through the system before birth. If the office of the placenta were like that of the lung, it cannot he supposed that the circulation of the resulting blood through the brain, and the whole system of the fœtus, would be compatible with the continuance of its existence as an embryo. We see that peculiar phase of existence terminate instantaneously with the development of arterial blood by the action of atmospheric air; and it would appear to be absolutely necessary, in order to preserve embryotic existence, that the blood of the embryo should be, perhaps, still farther removed from the arterial condition than the venous blood of the self-living animal.

It is contradictory to sound theory to suppose, that the placenta ought to supply the place of the lungs, or that any organ is wanted for their office, which is not only not required during fœtal life, but would be destructive of it. The same remark applies to the supposition, that the arterial blood of the mother acts upon the fœtal blood, similarly to the action of atmospheric air upon the blood in extra-uterine life, and it is contrary to fact to assert, that any change of the fœtal blood occurs in the placenta, similar to what occurs in the lungs during respiration. On the contrary, the condition of the fœtus bears an analogy to that of an hybernating animal in a state of winter sleep, and the mechanism of the placental vessels seems calculated to retard and dearterialize the maternal blood before its imbibition into the fœtal vessels, pretty much as the tortuosity of the arteries going to the brain in hybernating animals, and the vascular plexuses of tardigrade animals, are considered to be designed for an analogous object, and by a somewhat analogous mechanism. But however this may be, it is certain that the first development of independent existence, and the first manifestations of mind, are coincident with an alteration in the quality of the blood, and with a chemical change in that fluid. It is indeed doubtful, how the first inspiration is effected, but it may be suggested, that there is no necessity for supposing a single vital action, or a singular muscular movement, necessary for the first developement of that chemical change in the blood, which endows it with the stimulating properties that launch the whole animal organism into independent existence. For the contact of atmospheric air with any vascular membrane, is followed of necessity by the usual chemical reaction of such air on the blood contained in such membrane, and this blood thus changed necessarily excites pro tanto the vital action of whatever part it flows through, (independently of any influence from

* P. 320. † P. 139.

any central organ). Extra-uterine life may commence in the periphery, and extend to the centre, as death does sometimes. Besides, owing to the expansibility of gases, the atmospheric air, when brought into contact with the blood of any part, may operate on all the blood of the body, even before the mechanical movements of respiration have taken place (though very imperfectly indeed), yet sufficiently to impart to it the necessary stimulating properties. The circulation of the blood so acted on by the air, through the venous capillaries and trunks, is fully secured by that independence of the fœtal circulation on respiration which we see exemplified in the fœtus before birth. Thus, blood more or less arterialized may reach the brain before a single movement of the respiratory muscles has taken place, and, once that blood, even imperfectly arterialized, has come in contact with the nervous matter of the brain, will, motion, sensation, in a word, independent life commences.

The blood of the new born infant is remarkable for its small proportion of water, and for its larger proportion of globules, for the first few days after birth, perhaps even for the whole period during which the infant preserves the very rosy colour which is peculiar to it for two or three weeks. The liability of children to convulsions during this period, especially for the first eight days, is a subject of common remark. From two to five weeks to five months, the proportion of globules diminishes. Now, when we consider the apparent immunity which infants enjoy from derangements of the intellect, and their great liability to convulsions, we are led to think that the one takes the place of the other at this early age, and it has been argued by Dr. Prichard, and others, that convulsions and various mental derangements are allied diseases. This view is supported by the facts, that convulsions and mental derangements are frequently combined; that the same organ is the instrument of mind and of muscular motion; that the one affection frequently passes into some form of the other; that in families hereditarily predisposed epilepsy or convulsions of some other form will appear in one member, mania, melancholy, or some mental derangement in another; and that the same exciting causes appear sometimes to produce one or other of those affections indifferently. It should also be borne in mind that the acknowledged exciting causes of convulsions in new-born infants are, all of them, such as must materially affect the composition of the blood, as impure air, indigestion, the state of health of the mother, &c. During the early months of infancy, only faint traces appear of that intellect by which man is distinguished from all other animals; but from the first dawn of the reasoning faculties to adult age, a period passes in which the mental faculties, the temper, and the disposition, have a marked general character, and differ essentially in their qualities from those of adult and old age. During the whole period of man's life, we find the quality of the blood is altered in a slow and regularly graduated manner, according to the development of the intellect, the character of the temper, of the dispositions, of the affections, &c. M. Denis concludes, from his analyses, as related in M. Lecanu's essay, that from three weeks to five months the proportion of water increases, the proportion of globules diminishes.

From five months to about forty years the proportion of water diminishes, the proportion of globules increases.

From forty to seventy years the proportion of water increases anew, and that of the globules also anew diminishes.

Thus we have a large proportion of globules in the blood, coincident with the most perfect period of the intellect of man, and *vice versa*. Can changes in the solid nervous matter be demonstrated to occur in an equal degree, and with such a gradual correspondence? The adult time of life, the period of the highest physical and mental developement, the season of the passions, and of crime, but also of the most virtuous emotions and the most noble aspirations, is distinctly characterized by the highly organized condition of the blood, and it is at this period we find the greatest preponderance of red globules and of fibrine, in fact, of those portions of the blood which are known to exite most intensely and most permanently the nervous tissue. In old age—man's second infancy—we find the composition of the blood again approximating to its infantile condition, whilst great differences exist at those two periods of life in the activity of the circulation in the brain, and in its solid structure.

It is well known how much the intellect, the disposition, &c., of individuals is modified by their natural temperament, and it is found, that the relative composition of the blood varies according to the difference of temperament. For instance, the proportion of water is greater and the proportion of globules less in the lymphatic than in the sanguine temperament, and *vice versa.*

The constitution of the mind of man differs from that of woman, so also does the quality of their blood; the blood of women, like that of lymphatic individuals, having a larger proportion of water and a smaller proportion of globules.

In fine, on comparing the statistics in M. Quetelet's work on man, with the analyses of the blood given in M. Lecanu's essay, we can scarcely avoid being struck by the correspondence between the variations in the average mental and moral qualities of man, and the variations in the quality of the blood, as such variations are produced and modified by sex, age, food, climate, temperament, &c.

The necessity for the maintenance of life and consciousness, of the circulation in the brain, of blood more or less perfect, has been proved experimentally; and Richerand says:—"The speedy death of an animal is produced by tying the ascending aorta in a herbivorous quadruped, or at the same time, the vertebrals and carotids; and death is most probably to be attributed to the interruption of the circulation in the brain of arterial blood; because, if, the moment the vertebrals have been tied, the pipe of a syringe be adapted to them, and any fluid whatever is then injected with a moderate degree of force, and at nearly the same intervals as those of the circulation, life will not be restored." The same author remarks that "the energy of the brain appears to depend on the quantity of arterial blood which it receives." Muller admits the influence which the quality of the blood exercises on the manifestations of the mind, and adduces examples of it. The invigorating influence of exercise, pure air, and the moderate use of wholesome and nutritious food on the intellect are well known; and I will only remark, that the *modus operandi* of these agents is explainable in a more direct, palpable, and intelligible manner, on the supposition, that they act through their influence on the quality of the blood, and hence on the brain (a mode of action admitted for them by Müller), than by recourse to that obscure and mysterious power called sympathy.

The influence of hunger and repletion on the animal economy is extended to the mind, but their first influence is probably on the composition of the blood.

Inordinate passions, exertions of the intellect and moral emotions, are known to exert a powerful and often a permanent influence on the brain; but though it has long been known that some of those mental affections, when intense, alter the quality of the blood, yet this fact has been little if at all noticed in explaining the causes of the permanent effects upon the intellect, which sometimes follow. But as persons who die from fits of anger have their blood in a fluid and uncoagulated state, so it may be inferred that mental emotions of less intensity may effect minor changes in the blood, but yet sufficient to impair its healthy action on the brain. Keeping in mind the rule laid down by Müller,* and strictly supported by induction, viz: "That the physician has to keep in view, as the first point in all abnormal conditions of the mental functions, merely the nature of the structural change by which the action is rendered abnormal or prevented," it is much more philosophical to attribute the permanent derangements of intellect which follow sudden and violent mental emotions to some physical change in the animal economy than to any reflex

* P. 821.

action of those emotions upon the mind itself, which would involve the supposition (hard to be conceived), that the immaterial mental essence becomes diseased. *Post-mortem* examinations have not established the occurrence of lesions in the solid structure of the brain in cases of sudden death, or of mental alienation, arising from intense mental emotions. The rapidity of the effect in those cases accords much better with the idea of a change in a fluid, so complex, so delicate, and so susceptible of alteration as the blood, than it does with the idea of a change in the solid structure of a body, so permanently organized as that of the brain; whilst any temporary change in the mere amount of blood circulating in the brain, or in the rate or mode of its circulation, cannot well be conceived as an efficient cause, *per se*, of symptoms so varied and so permanent. We read in Pringle, that "scurvy broke out with increased virulence immediately on the receipt of disastrous news by the army whose medical management he superintended, without any change in the physical comforts or circumstances of that army."

Here, again, we witness mental emotions, adding to the virulence of a disease, which is chiefly characterized by a morbid condition of the blood. With the progress of analytical and experimental science, the wide range of influence over the animal economy, previously attributed to sympathy, and other vague, occult, and mysterious agents, has been gradually narrowed. Thus, a great number of poisons, which heretofore were considered as acting, some directly on the nerves and others through a mysterious and ill-defined sympathy of one organ over another, are now proved to enter the blood, and to act through the blood, sometimes on the brain, sometimes on the heart, sometimes on the stomach, &c. In fact, many poisons applied locally, and directly, exert little influence on the part to which they are applied; and, on the contrary, act powerfully when introduced into the blood. "Before narcotic poisons can exert their general effects on the nervous system," says Müller, "they must enter the circulation. It is still, however, very common to attribute nervous disturbances arising in the course of affections of important organs, as of the stomach, liver, heart, lungs, uterus, &c., to the effects of sympathy on the part of the brain with those organs. I do think that even what remains of the doctrine of sympathy is carried to a greater length than is supported by facts, or rational induction. The doctrine and principle of sympathy, modified as it is at the present day, is quite unequal to the explanation of the infinite variety of the so-called sympathetic affections of the brain. Under such a doctrine the depraved tastes of chlorosis, and the fitful moods of hysteria, the morbidly clear intellect of cholera (which evinces no sympathy with a disease, the aspect of which strikes terror into the mind of all but the subject of it), the despondence, the delirium, or the coma of jaundice, the hypochondriasis of gastric dyspepsia, the active delirium of gastritis, the buoyant hopefulness of phthisis, and the oppressed brain of double bronchitis, become a tangled and inextricable web. On the other hand, we know that the blood is altered in many, perhaps we may truly say in every bodily disease which has a name in our nosology, and the changes which occur in the tone and temper of the mind and feelings in those diseases, seem capable of rational explanation, most simply and most naturally on the supposition of the reaction which the blood exerts on the nervous matter of the brain, according to its quality. How simple—how natural—how consistent with known facts is the notion, that the sympathies of the brain with various organs are effected through that vital fluid, the blood, which is changed in its qualities by every organic function of the body, and which, in its turn, reacts upon the nervous system! That delicacy, infinite diversity, and rapidity of change, of which a complex fluid like the blood is so much more susceptible than any solid, seem alone capable of explaining the minute shading of intellect, of character, and of feelings—the rapid alternation from one extreme of feeling and of thought to another, and the vast diversity of the human mind, whether in health or disease. The permanence and the sameness of form which distinguish solid structure, seem incompatible with such qualities. In the laughing gas, in intoxicating liquors, and in many of the narcotic poisons especially, we have familiar instances of the action of organic and inorganic matters mixed with the blood, in producing a great variety of allied, and yet distinct impressions on the intellect and moral feelings. The strong propensity to laughter, and rapid flow of vivid ideas produced by the inhalation of nitrous oxide gas—the muddled mirth arising from ale and porter—the sparkling wit which circulates with the flowing juice of the grape—the frenzy, half-joyous, half-quarrelsome disposition, which are the effects of whisky-drinking—the blissful delirium of the opium-eater—can vague sympathies—can altered combinations of solid molecules—can variations in the quantity, the mode, or the rate of the circulation of the blood in the brain—can any, or all of those in themselves, explain the minute, the endless variety of the modes of thought and feeling of which the above are a few instances? There are solid substances, no doubt, which produce the same effects on the brain as fluids, but to do so they must first be dissolved, and enter the blood. Then, and not until then, is the brain affected. In the action of narcotic poisons introduced into the stomach, not the least difference can be perceived, according to Müller, whether the nervous vagus has been divided or not. In like manner, certain descriptions of food affect the mind in specific modes, either owing to idiosyncrasies or as a normal law.

As the various organic and inorganic substances introduced into the blood *ab extra*, affect the brain in certain specific ways, what reason is there for supposing that organic, and inorganic substances, developed *ab intra* in the animal economy and circulating in the blood, will not exert also a specific action according to the properties of each? And what is this but the exercise of an influence, on the part of the blood, according to its qualities, over the functions of the brain, as a normal law of the animal economy? A healthy brain, acted on by healthy blood, produces healthy action, yet modified within certain limits, by the relative proportions, or more or less perfect organization of the natural constituents of the blood. And in diseased action of the mind, may we not have an abnormal constitution of the blood, owing to extraneous matters mixed therein, incompatible with the sane action of the brain? The various bodily functions and secretions are rarely, if ever, duly discharged, during, or even prior to, derangements of mind. Defective assimilation and elimination must vitiate the blood with crude or effete matter, and this matter may, of itself or in new combinations, form the morbid cause of insanity in many instances. The constituents of nitrous oxyde gas, for instance, exist largely in the food and blood of man, and the developement of this gas, which exercises such a powerful influence over the nervous system, is not impossible in the human body. And if this particular gas be not developed, yet there may be formed other equally potent bodies, whether fluid or gaseous, which, mixed with the blood, may exercise a sway equally uncontrollable over the human brain—when the bodily functions, from whatever cause, are not duly discharged, when bodily disease, however produced, is present, the elements, and more particularly the fluid elements of the individual organism whose functions are so deranged, and whose body is so diseased, are more or less released from the control of the laws peculiar to the specific organization of that individual, and fall more or less, perhaps, under the laws of some different type of animal organization, or even under those of vegetable life, or of inorganic matter. The vegetative formations which sometimes occur on the skin, particularly of persons fed on insufficient or unwholesome food, tumours and other morbid products, the putrid condition of the blood in scurvy, as well as in other diseases, appear to be extreme instances of such a vital degeneration. But how many abnormal compounds, how many new chemical combinations foreign to its normal constitution and to its healthy action, may arise in the blood, when the sway of the vital laws has been loosened, and when this complex fluid comes, even very partially, under the influence of the chemical laws of inorganic matter, or the vital laws of lower types of organism? And what may be the influence of those new compounds and combinations on the brain? Those questions are suggestive of a field of observation as yet scarcely entered upon. The length to which those remarks have already run prevents my entering into a detailed investigation of the state of the nervous functions, in various bodily diseases in which an alteration of the quality of the blood is known to exist, and in which also the nature of that alteration has been at least partly ascertained.

I do not suppose that by such an investigation I could establish, in an unquestionable manner, that there exists any constant and definite dependence of one upon the other. The chemical analysation of the blood is perhaps as yet in too imperfect progress to expect such a result. Still less do the materials exist for drawing unquestionable inferences from the quality of the blood in purely mental diseases. In those so called purely mental affections little indeed is analytically known of the state of the blood. But I may say that what is known of the state of the blood in bodily and mental disease, tends to establish the *prima facie* inference at least, which I have advocated chiefly on physiological reasons in the foregoing pages. I may add, that even from my own limited observations on the quality of the blood, and its influence over the intellectual and moral functions, both in bodily and mental disease, I could put forward some considerations in support of the foregoing views, and at some future period I may, perhaps, venture to do so. On the other hand, I have not made one single observation contradictory of those views. But for the present, if I only succeed in directing a closer attention to the composition of the blood in mental affections, whether supposed to be pure or complicated with bodily ailments, or if I call forth the results of such observations, by parties better qualified than I am for the task, I shall have attained my chief object. Many of the points to be investigated, as directly or indirectly in connexion with this subject, will at once suggest themselves to all. Yet as it might somewhat facilitate the inquiry, to have all the chief points at least to be investigated, and the mode and means of investigating them brought at one view before the profession, it would be very desirable if a definite scheme or plan for such an inquiry were put forward by some party whose attention has been directed to this subject, and to the chemical details and manipulations connected with it. In our public hospitals for the treatment of the insane, all the combinations necessary for the successful prosecution of such an inquiry either already exist, or could be easily brought together.

